# Addressing barriers to equitable telehealth for older adults

**DOI:** 10.3389/fmed.2025.1483366

**Published:** 2025-01-23

**Authors:** Bruce Leff, Christine S. Ritchie, Kristin L. Rising, Kendell Cannon, Liane Wardlow

**Affiliations:** ^1^Department of Medicine, Division of Geriatric Medicine and Gerontology, Johns Hopkins University School of Medicine, Baltimore, MD, United States; ^2^Massachusetts General Brigham Department of Medicine, Harvard Medical School, Massachusetts General Hospital, Boston, MA, United States; ^3^Center for Connected Care, Thomas Jefferson University, Philadelphia, PA, United States; ^4^Primary Care and Population Health, School of Medicine, Stanford University, Stanford, CA, United States; ^5^Clinical Research, West Health Institute, La Jolla, CA, United States

**Keywords:** telehealth, equity, older adults, telemedicine, ageism

## Background

The number of older adults (65+) in the United States grew by 34% between 2012 and 2022. By 2040, more than 78 million Americans will be 65 or older, which is more than double the number (35 million) of people in that age category in 2000 (1). Aging is associated with the development of chronic conditions and multimorbidity (2), which led older adults to incur, on average, $5,277 in medical expenses in 2022 despite only having a median income of $29,740. Although the US general population spends approximately 8% of its total expenditures on health, older Americans spend approximately 13% of their total expenditures on health (1).

As the number of older adults is growing, the number of people living with multiple chronic conditions is also growing (3). Those with more chronic conditions tend to have fewer financial resources and greater physical limitations, often making it difficult to get to medical appointments (4, 5). Telehealth is a particularly useful tool in helping manage many chronic conditions, particularly when it can be delivered at lower cost to patients and reduce the need for physical travel (6).

In the wake of the COVID-19 pandemic, telehealth technology grew tremendously, particularly in its usage to manage chronic conditions of older adults who were particularly susceptible to harmful effects of COVID-19 but who continued to need ongoing medical care (7). Telehealth, the use of technology to facilitate encounters and/or the exchange of medical information between providers and patients at a distance, can enable remote consultations, diagnoses, and patient monitoring using video conferencing, mobile apps, and wearable devices, as examples. Telehealth, which is generally accepted as a broader term than the term telemedicine, can also include patient and provider education, patient self-management support, and health information services (8). Telehealth offers benefits including increased access to timely care, reduced patient travel burdens, improved efficiency for physicians, and early intervention in critical situations (9, 10). Advancements in telehealth technology have paved the way for a future where healthcare services are readily available to everyone, irrespective of geographical barriers, age, and health status. In this paper, we delineate barriers and biases to the equitable use of telehealth to care for older adults and approaches to identify and ameliorate such barriers (11).

## Barriers to equitable use and uptake of telehealth

Although the benefits of telehealth are clear, certain people, including some older adults, may experience barriers to its utilization creating a potential for inequity in care delivery. Research shows that many older adults face unique challenges to the adoption of telehealth that include physical and cognitive challenges associated with aging, low health literacy, technology-related challenges associated with lack of access, low technology literacy, discomfort with new technologies, and implicit ageist biases, any of which may prevent health care providers from offering them telehealth services.

Barriers associated with aging include cognitive and sensory impairments and reliance on caregivers or family members to assist with technology (12, 13). In one study, 82% of homebound patients (mean age 82.7; 46.6% with dementia) required assistance from a family member and/or paid caregiver to complete a telehealth visit (14). Another study found that older adults with visual impairments were less likely to have or know how to use a cell phone, a tablet, or a computer. Lack of access to technology can also be a barrier for older adults (15). Although access is increasing, only 61% of adults 65 years and older were using smartphones in 2021 (16). Additional barriers related to technology include device specific complaints such as difficulty learning to use devices, costs of devices, lack of instruction, and technical support, and technology anxiety and technophobia, among others (17, 18).

An additional set of barriers comes in the form of biases that prevent telehealth use among older adults including ageism, ableism, and the infantilization of many older adults (19). Implicit biases are associations made outside of conscious awareness leading to a negative evaluation of a person that are based upon irrelevant characteristics such as age or gender (16, 20). Research shows that physicians are not immune to implicit biases, and in fact hold the same level of implicit bias as the rest of the population (20). Importantly, evidence suggests that these biases can negatively impact diagnoses and treatment decisions (21). Although true intrinsic difficulties to technology use are present with increasing frequency in older adults, the bias that all older adults “can't use technology,” “can't hear,” or “can't see” has created a paternalistic attitude toward older adults' capabilities (22). As with all healthcare, a clear look at identifying underlying biases, including cultural, socioeconomic, racial, and educational biases, will enable us to identify and tailor care directly to individual patients rather than generalizing across a population.

## Identification of and approaches to addressing barriers

A key step to address barriers to equitable use of telehealth for older adults is to first identify the specific barrier(s) a patient may be facing. Ideally, systematic screening at the level of the health system, and taking a multi-stakeholder approach to identifying barriers (23) can be implemented to identify barriers at the individual and population levels and so that those identified barriers can be matched with available interventions. At a minimum, initial assessments should include understanding what technologies patients have access to (smartphone, broadband, computer), digital literacy (skills needed to perform tasks online or on computers), and health literacy (skills to identify, understand, and use health-related information), which tend to be associated with one another (24). The Telehealth Literacy Screening Tool is one example of such an assessment that can be used with older adults (25). Additional relevant factors include implicit age-related biases in health care delivery. Validated instruments to assess one's own implicit biases such as the Implicit Association Test can be used to uncover and serve as an initial step to address age-related biases (26).

As telehealth is increasingly used with and by older adults, effort should be directed to assure that it is purposefully designed to address older adults' needs. Options should include accommodations for users with visual impairment, by offering digitally enlarged text, visual optimization of printed materials, and instructional guides available in audio and text versions. To address discomfort with new technologies, services should use platforms and interfaces that older adults already comfortable with using, to the extent that this can be generalized (27). Telehealth should incorporate human-centered design strategies and be developed in collaboration with older adults. Systematic assessments of populations to understand the prevalence of digital health readiness barriers can inform prioritization of interventions at the system level, and deployment of the right interventions to the right individuals. We describe several existing exemplar programs that address a variety of barriers, along with suggestions for expansion where applicable.

### Technology and broadband access

Programs that provide discounted devices and broadband access, mostly with income caps, are available, including Comcast's Affordable Connectivity Program. Though these programs help some “at need” populations, more work is needed to ensure everyone who is eligible knows about and can access these services. Deployment of individuals such as community health workers into communities to provide active outreach both informing and enrolling people into these programs may improve access.

### Access hubs

Hubs provide a centralized location for patients to come to use digital resources. These hubs can assist individuals who have unstable housing or unreliable broadband access to more reliably access devices and the internet. Hubs can also benefit older adults who need more technical support as staff can be stationed there to provide face-to-face, real-time support. A variety of community-based programs often function as an access hub including local libraries and community centers.

### Facilitated technological support

Some organizations have evaluated various facilitation methods including a change in service design to address intrinsic barriers to independent technology use, such as cognitive or functional limitations. Making models in which support individuals can facilitate telehealth visits for older adults available, including sending emergency medical technicians (EMTs) or community health workers to patients' homes or using staff facilitators in long-term care facilities, is vital (28).

### Language and culture considerations

Cultural barriers to telehealth use for older adults must also be addressed. In a consensus conference focused on developing a patient-centered research agenda to reduce disparities in telehealth uptake “addressing cultural facilitators and barriers to telehealth use” was identified as one of the top three priorities (29). Studies support the need for increased focus on cultural competency, specifically for populations with limited English proficiency (30). A study of medical students documented lower confidence in providing care to patients with limited English proficiency via telehealth as compared to in-person (31). Moreover, work has documented challenges in incorporating translator services into telehealth (32).

### Training of providers

Providers also require specific training focused on recognizing and overcoming their own biases about telehealth and who is “appropriate for” or able to engage in a telehealth visit, particularly with respect to older adult patients. Increasingly, health care organizations are investing in implicit bias trainings (33). Instead of wondering “who,” clinicians must be trained to think “how” to facilitate a telehealth visit for anyone, thus ensuring the same access to a valuable component of care.

## Conclusion

As telehealth becomes a ubiquitous part of healthcare, identification of barriers to older adults' usage of telehealth and systematic strategies to address them can reduce healthcare inequities and potentially improve access and quality of life for a growing number of older adults. While we focus on strategies for identifying and mitigating barriers to telehealth utilization, we must also ensure we consider implicit biases that may prevent providers from offering telehealth to older adults.

## Author's note

We certify that this work is novel.
